# TENERGY: multicenter phase II study of Atezolizumab monotherapy following definitive Chemoradiotherapy with 5-FU plus Cisplatin in patients with unresectable locally advanced esophageal squamous cell carcinoma

**DOI:** 10.1186/s12885-020-06716-5

**Published:** 2020-04-20

**Authors:** Hideaki Bando, Daisuke Kotani, Takahiro Tsushima, Hiroki Hara, Shigenori Kadowaki, Ken Kato, Keisho Chin, Kensei Yamaguchi, Shun-ichiro Kageyama, Hidehiro Hojo, Masaki Nakamura, Hidenobu Tachibana, Masashi Wakabayashi, Miki Fukutani, Yosuke Togashi, Nozomu Fuse, Hiroyoshi Nishikawa, Takashi Kojima

**Affiliations:** 1 Department of Clinical Oncology, Aichi Cancer Center Hospital, Nagoya, Japan; 2 Department of Gastroenterology and Gastrointestinal Oncology, National Cancer Center Hospital East, Kashiwa, Japan; 3 Division of Gastrointestinal Oncology Shizuoka Cancer Center, Shizuoka, Japan; 4 Department of Gastroenterology, Saitama Cancer Center, Saitama, Japan; 5 Department of Gastrointestinal Medical Oncology, National Cancer Center Hospital, Tokyo, Japan; 6 Department of Gastroenterological Medicine, Cancer Institute Hospital of Japanese Foundation for Cancer Research, Tokyo, Japan; 7 Division of Radiation Oncology and Particle Therapy, National Cancer Center Hospital East, Kashiwa, Japan; 8 Clinical Research Support Office, National Cancer Center Hospital East, Kashiwa, Japan; 9 Division of Cancer Immunology, Exploratory Oncology Research and Clinical Trial Center, National Cancer Center Hospital East, Kashiwa, Japan

**Keywords:** Unresectable locally advanced, Esophageal squamous cell carcinoma, Chemoradiotherapy, Atezolizumab

## Abstract

**Background:**

The standard treatment for patients with unresectable locally advanced esophageal squamous cell carcinoma (ESCC) is definitive chemoradiotherapy (CRT) using 5-FU plus cisplatin. However, complete response (CR) rates are low at 11–25%, resulting in 9–10 months of median overall survival (OS). An improved therapeutic efficacy by combining immunotherapy with radiation has been reported in patients with locally advanced non-small cell lung cancer. The results using ESCC cell lines suggest sequential treatment with anti-PD-L1 agents soon after completion of CRT is the most effective combination.

**Methods:**

TENERGY trial is a multicenter, phase II, proof-of-concept study to assess the efficacy and safety of atezolizumab following definitive CRT in patients with locally advanced ESCC. The main inclusion criteria are unresectable locally advanced ESCC without distant metastasis, completion of 60 Gy of radiation plus two concomitant cycles of chemotherapy (cisplatin 70 mg/m^2^ on day 1 and 5-FU 700 mg/m^2^ on days 1–4, every 28 days), and adequate organ function. Within 6 weeks after CRT, participants will start taking 1200 mg of atezolizumab every three weeks and continue until 12 months or disease progression. The primary endpoint is the confirmed CR rate by the investigator’s assessment. Secondary endpoints include overall response rate, progression-free survival (PFS), OS, adverse events, and confirmed CR rate by central assessment. We will enroll 50 patients (40 with primary locally advanced ESCC and 10 with postoperative locoregionally recurrent ESCC). We will obtain biopsies from the primary site and will collect blood at 3 time points (before CRT, after CRT, and four weeks after the start of atezolizumab) for an exploratory biomarker study. We will analyze the phenotype of immune-competent cells, neoantigens, tumor mutational burden, PD-L1 status, and Human Leukocyte Antigen haplotyping.

**Discussion:**

The synergistic efficacies of the sequential combination of CRT and atezolizumab should improve the CR rate, resulting in survival improvement for patients with unresectable locally advanced ESCC. Because CRT is a standard treatment option for patients with early stage to locally advanced ESCC, the sequential combination of CRT and atezolizumab has the potential to change the standard ESCC treatments.

**Trial registration:**

UMIN000034373, 10/04/2018 and EPOC1802.

## Background

Carcinoma of the esophagus is an extremely devastating disease, especially when the disease invades adjacent structures such as aorta, vertebral bodies, or trachea (T4b), and becomes unresectable. According to the Comprehensive Registry of Esophageal Cancer in Japan, the incidence of T4b esophageal cancer accounts for approximately 6.7% of all patients with esophageal cancer (approximately 1500 patients per year) [1]. The standard treatment for this population is definitive chemoradiotherapy (CRT) using 5-FU plus cisplatin. However, complete response (CR) rates are low at 11 to 25%, resulting in 9 to 10 months of median overall survival (OS) [2–4]. Although new strategies have been investigated [4], the treatment regimens have not changed since 1990s.

Immunotherapy with immune checkpoint inhibitors (ICIs) has revolutionized the treatment of advanced cancers, including that of esophageal cancer. Pembrolizumab, an anti-programmed death 1 (PD-1) antibody, significantly improved OS in patients with programmed death ligand 1 (PD-L1) combined positive score (CPS) ≥10 metastatic esophageal cancer [5]. Subgroup analyses indicated higher efficacies of pembrolizumab for patients with esophageal squamous cell carcinoma (ESCC) than those for patients with adenocarcinoma, and the Food and Drug Administration (FDA) approved pembrolizumab for patients with metastatic ESCC whose tumors express PD-L1 CPS ≥10 after ≥1 prior line of systemic therapy. Subsequently, nivolumab, another anti-PD-1 antibody, showed significant OS improvement in patients with metastatic ESCC after ≥1 prior line of systemic therapy (regardless of PD-L1 status) [6].

ICIs combined with ionizing radiation are promising approaches due to their efficacies. Mechanisms facilitating the action of ICIs by radiation include increased tumor antigen release, activation of innate immune pathway, increased T-cell infiltration, augmented antigen presentation, and modulation of immunosuppressive cells [7, 8]. Indeed, in in vivo models, sequential combination of an anti-PD-1 antibody and radiation increased the proportion of tumor antigen complexes and major histocompatibility complex (MHC) molecules, enhanced lymph node cross-presentation, and increased T-cell tumor infiltration [9]. The polyclonal T-cell response also mediated out-of-field (abscopal) effects following local radiotherapy [10]. An abscopal effect from the combination of radiation and immunotherapy has also been reported in cases with different cancer types [11]. Phase I trials showed a 10–13.5% response rate for liver or lung metastases outside the radiation field [12, 13]; thus, similar efficacies may be expected for micro metastatic lesions in patients with locally advanced cancer.

According to other studies, chemotherapy and radiotherapy may mediate the release of interferon gamma (IFN-γ) produced by CD8+ T cells resulting in PD-L1 upregulations in various tumor cells [8, 14]. Our preliminary studies using both ESCC cell lines and a radiation irradiation device used in the clinical practice also reported that 60 Gy of radiation upregulated only the expression of PD-L1 and MHC Class I without affecting the expression of PD-L2 (data not shown). As lymphocytes are radiation sensitive, we hypothesized that the sequential treatment with anti-PD-L1 agents soon after completion of CRT would enhance the treatment efficacies.

Among patients with unresectable locally advanced non-small cell lung cancer, 12 months of the anti-PD-L1 antibody durvalumab following platinum-based CRT significantly improved both PFS and OS irrespective of PD-L1 expression before CRT [15, 16]. Durvalumab also had a favorable effect on the frequency of new metastases, including on the incidence of new brain metastases. The safety profile of durvalumab was consistent with those of other immunotherapy-related trials, and included a relatively high but acceptable radiation or ICI-induced pneumonitis rate.

Based on this information, we have planned a phase II proof-of-concept (POC) clinical trial to evaluate the safety and efficacy of the anti-PD-L1 antibody atezolizumab following definitive CRT in patients with unresectable locally advanced ESCC.

## Methods/design

### Study design and treatment

**Fig. 1:**
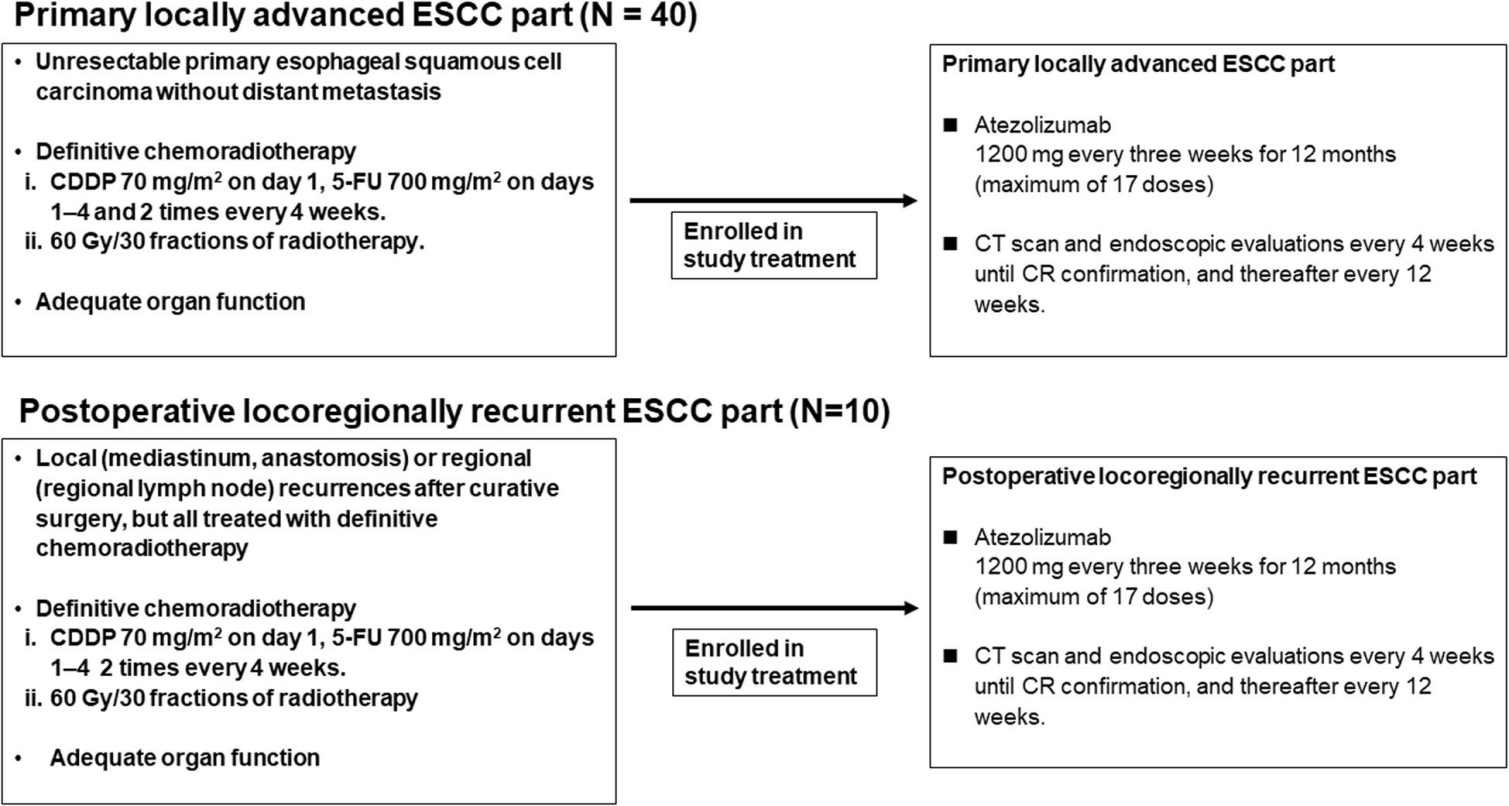
Study design. The TENERGY trial consists of two parts. After completion of definitive chemoradiotherapy, patients with primary locally advanced esophageal squamous cell carcinoma (ESCC) will be enrolled into the primary locally advanced ESCC part (*N* = 40) Patients with locoregionally recurrent ESCC after surgical resection will be enrolled into the postoperative locoregionally recurrent ESCC part (*N* = 10)

The TENERGY trial is a multicenter, phase II POC study to assess the safety and efficacy of a sequential combination therapy with atezolizumab following 5-FU plus cisplatin-based chemoradiotherapy (60 Gy/30 fractions without prophylactic irradiation) in patients with unresectable locally advanced ESCC without distant metastasis. This study consists of two parts: we will enroll patients with primary locally advanced ESCC into the primary locally advanced ESCC part, and patients with locoregionally recurrent ESCC after surgical resection into the postoperative locoregionally recurrent ESCC part (Fig. 1).

Table 1 shows the inclusion and exclusion criteria for both parts. We will provide the intervention treatment to patients who meet all the inclusion criteria and do not meet any of the exclusion criteria. Although the CRT is not included in the protocol therapy, both chemotherapy and radiation will be stringently qualified based on the quality assurance and quality control program. We will initiate the study intervention treatment within 6 weeks after CRT completion. Patients will receive 1200 mg of atezolizumab every three weeks until 12 months (maximum of 17 doses), disease progression, patient withdrawal, investigator’s decision, pregnancy, or unacceptable toxicities (whichever comes first).

**Table 1:** Patient inclusion and exclusion criteria

Inclusion Criteria	
Inclusion Criteria (for the primary locally advanced ESCC part)	
1. Patients with histologically confirmed primary esophageal squamous cell carcinoma, adenosquamous carcinoma, or basaloid-squamous carcinoma.	
2. Patients with primary lesion located in the thoracic esophagus based on the UICC TNM 7th edition classification, patients with invasion to cervical esophagus and/or abdominal esophagus are eligible when definitive CRT is eligible, patients with secondary lesions that are curable by endoscopic resection are eligible.	
3. Patients diagnosed as having an unresectable tumor by a pre-CRT CT scan as described below. Patients with esophageal perforation, esophagotracheal fistula, esophagomediastinal fistula, bleeding from arterial invasion, or respiratory stenosis are ineligible.	
i. The depth of the primary tumor is diagnosed as T4b (tumor invades other adjacent structures such as aorta, vertebral body, or trachea) according to the UICC TNM 7th edition and Japanese Classification of Esophageal Cancer The 11th Edition.	
ii. Regional lymph nodes and/or supraclavicular lymph node have invaded adjacent structures other than esophagus.	
4. Patients without distant metastases other than supraclavicular lymph node by pre-CRT CT scan.	
5. Patients without prior esophageal cancer treatment except for endoscopic resection before CRT.	
6. Patients for whom the CRT described below has been performed and for whom the protocol treatment can be started within 42 days after CRT completion.	
i. Chemotherapy started at CDDP 70 mg/m^2^ on day 1, 5-FU 700 mg/m^2^ on days 1–4 and 2 times every 4 weeks. Second cycle dose reductions were allowed according to the toxicities.	
ii. 60 Gy/30 fractions of radiotherapy performed without exceeding dose limitations to at-risk organs.	
7. Patients without esophageal perforation, esophagotracheal fistula, esophagomediastinal fistula, bleeding from arterial invasion, or respiratory stenosis when CRT is completed.	
Inclusion Criteria (for the postoperative locoregionally recurrent ESCC part)	
1. Patients with histologically confirmed esophageal squamous cell carcinoma, adenosquamous carcinoma, or basaloid-squamous carcinoma on surgical specimens.	
2. Patients with local (mediastinum, anastomosis) or regional (regional lymph node) recurrences after curative surgery, whose recurrences are considered treated with definitive CRT.	
3. Patients without distant metastases except those in supraclavicular lymph nodes by pre-CRT CT scan.	
4. Patients treated with neoadjuvant chemotherapy 24 months or more before start-up of any definitive CRT.	
5. Patients for whom the CRT described below has been performed and those for whom the protocol treatment can be started within 42 days after CRT completion.	
i. Chemotherapy started at CDDP 70 mg/m^2^ on day 1, 5-FU 700 mg/m^2^ on days 1–4 and 2 times every 4 weeks. Second cycle dose reductions were allowed according to the toxicities.	
ii. 60 Gy/30 fractions of radiotherapy performed without exceeding dose limitations to at-risk organs.	
6. Patients without esophageal perforation, esophagotracheal fistula, esophagomediastinal fistula, bleeding from arterial invasion, or respiratory stenosis when CRT is completed.	
Inclusion Criteria (common to both study parts)	
1. Patients 20 years or older.	
2. Patients with Eastern Cooperative Oncology Group Performance Status (ECOG PS) 0 or 1.	
3. Patients with the following organ functions within 14 days of registration:	
i. Neutrophil count ≥1200/mm^3^	
ii. Platelet count ≥75,000/mm^3^	
iii. Hemoglobin ≥8.0 g/dL	
iv. Serum albumin ≥2.5 g/dL	
v. T-Bil ≤1.5 x ULN, in the case of Gilbert’s syndrome T-Bil ≤2.5 x ULN	
vi. ALT and AST < 100 IU/L	
vii. Serum creatinine ≤1.5 mg/dL or calculated or measured values of creatinine clearance ≥45 mL/min	
viii. SpO2 ≥ 95% (room air)	
4. Women of childbearing potential with a negative urine pregnancy test within 14 days of registration.	
5. Patients who agree to take appropriate precautions to avoid pregnancy from the time of the informed consent to 5 months after the final administration of the investigational drugs.	
6. Patients with life expectancy longer than 3 months.	
7. Patients who sign written informed consent.	
Exclusion Criteria	
1. Patients with active multiple primary cancers (synchronous multiple primary cancers and multiple cancers with a progression-free period of ≤3 years from the time of enrollment), carcinoma in situ or intramucosal carcinoma curable by local resection are not included in the definition of active multiple primary cancers.	
2. Patients with active infection for which systemic treatments are needed.	
3. Patients with past or concurrent inflammatory bowel disease.	
4. Patients with past or concurrent pneumonitis or interstitial lung disease (ILD).	
5. Patients with concurrent autoimmune disease or with past chronic or recurrent autoimmune disease.	
6. Patients requiring treatment with systemic corticosteroids or immunosuppressants.	
7. Patients with past or concurrent thyroid dysfunction (hyperthyroidism or hypothyroidism).	
8. Patients with a history or finding of a cardiovascular risk:	
i. Patients with a left ventricular ejection fraction below 50%	
ii. Patients with clinically significant, poorly controlled arrhythmia	
iii. Patients with acute coronary syndrome, coronary angioplasty, or stent placement within 6 months before enrollment	
iv. Patients with class II or more severe congestive heart failure	
v. Patients with treatment-resistant hypertension	
vi. Patients wearing an implantable cardioverter defibrillator or permanent pacemaker	
9. Patients with poorly controlled diabetes mellitus.	
10. Patients positive for HIV antibody, HBs antigen or HCV antibody.	
11. Pregnant or lactating patients.	
12. Patients with a significant unstable mental disease or other medical disease that may interfere with their safety, obtaining informed consent, or compliance with the procedures for the clinical study.	
13. Patients with a history of treatment with atezolizumab, anti-PD-1 antibody, anti-PD-L1 antibody, anti-PD-L2 antibody, anti-CD137 antibody, or anti-CTLA-4 antibody or a history of any other antibody therapies or drug therapies intended to regulate T cells.	
14. Patients who are not willing to or who cannot comply with the procedures specified in the protocol.	
15. Patients unsuitable for the study due to complicated disorders affecting the assessment of toxicity according to the investigator’s judgment.	

We are conducting this study in accordance with the guidelines for Good Clinical Practice of the International Council on Harmonization of Technical Requirements for Registration of Pharmaceuticals for Human Use, as well as with the ethical guidelines for medical and health research involving human subjects. All patients are required to sign written informed consents. We registered the study in the University Hospital Medical Information Network (Clinical trial information number: UMIN000034373).

### Endpoints and assessments

The primary endpoint is the confirmed CR rate by an investigator’s assessment in the primary locally advanced ESCC part. The secondary endpoints are the confirmed CR rate by central assessment, the objective response rate (ORR) by the investigator’s assessment, the PFS, the OS, and the incidence of adverse events (AEs).

CR will be determined by both computed tomography (CT) scanning and endoscopy based on the Response Evaluation Criteria in Solid Tumors (RECIST) version 1.1 with modifications and on the Japanese Classification of Esophageal Cancer (11th edition), respectively. The schedule for both CT scans and endoscopy examinations are every 4 weeks until CR, following the confirmation of CR after more than 4 weeks, and thereafter every 12 weeks. The modified RECIST defines both measurable and non-measurable lymph nodes as those ≥10 mm and 5–10 mm in their short axis, respectively, when assessed by CT scan. According to the Japanese Classification of Esophageal Cancer, we defined the primary lesion is as CR when conditions satisfy all of the following 4 factors: disappearance of endoscopic findings suggesting the presence of a tumor, negative endoscopic biopsy findings from the area of the primary tumor, evaluable entire esophagus using endoscopy, and no endoscopic findings of active esophagitis. We defined ORR as the proportion of patients who achieve CR or partial response (PR). We defined PFS as the period from registration to progression or death from any cause and will censor it on the last day the patient is alive without progression. OS is the period from registration to death from any cause, and we will censor it on the last day the patient is alive. We will assess AEs according to the Common Terminology Criteria for Adverse Events (CTCAE) version 4.0 before administration of the investigational drug on the administration day. In the postoperative locoregionally recurrent ESCC part, we will assess the same endpoints in an exploratory manner.

### Target sample size and statistical analyses

The reported CR rates have ranged from 11 to 25%. In this study, we estimate a rate of 20% as the CR threshold. Therefore, we calculated the sample size of the primary locally advanced ESCC part at 38 with a CR of 40% deemed promising and one of 20% deemed unacceptable (one-sided α, 0.05; β, 0.2). Under this calculation, we are planning to include a maximum of 40 patients in the primary locally advanced ESCC part. We set the planned sample size of the postoperative locoregionally recurrent ESCC part at a maximum of 10 patients in an exploratory manner. We will calculate the confirmed CR by the investigator’s assessment and its 90% confidence interval using the exact binomial method. The results will be considered statistically significant with more than 13 confirmed CR cases in accordance with both the RECIST and the Japanese Classification of Esophageal Cancer in the primary locally advanced ESCC part (i.e., CR rate ≥ 34.2%). We will also determine the confirmed CR rate by central assessment, the ORR by the investigators’ assessment, the PFS, and the OS using appropriate statistical methods. Finally, we will also tabulate the incidence of AEs in the safety population.

### Biomarker analyses and translational research

We will perform serial biopsies from the primary site and blood collections at 3 time points (before CRT, after CRT, and four weeks after the first atezolizumab dose). Using the collected samples, we will investigate biomarkers for efficacy or resistance to the sequential combination of CRT and atezolizumab. We will also analyze whole exome sequencing, neoantigens, microsatellite instability, tumor mutational burden, phenotype of immune-competent cells using both flowcytometry and immunohistochemistry, PD-L1 status, and Human Leukocyte Antigen haplotyping.

## Discussion

The TENERGY trial is the first phase II POC study to evaluate the efficacy and safety of sequential combination therapy with 1 year of atezolizumab following definitive CRT in patients with unresectable locally advanced ESCC. The synergistic efficacies of CRT plus ICIs combination should improve the confirmed CR rate, resulting in a prolonged survival. If the combination significantly improves the confirmed CR rate, we will plan a confirmatory phase III trial. Also, as the incidence of T4b esophageal cancer is estimated approximately at 1500 patients per year in Japan, we expect the Japanese Accelerated Approval Program will allow for earlier approval of drugs that treat serious conditions, and that fill an unmet medical need.

Moreover, because CRT is one of the standard treatment options for patients with early stage to locally advanced ESCC, the application of sequential combinations of CRT plus ICIs should be potentially expandable to patients with all ESCC stages and the results of our trial may guide standard ESCC treatment modifications.

## Data Availability

The datasets used and/or analyzed during the current study are available from the corresponding author on reasonable request.

## Abbreviations

CRT: Chemoradiotherapy
CR: Complete response
OS: Overall survival
ICIs: Immune checkpoint inhibitors
PD-1: Programmed death 1
PD-L1: Programmed death ligand 1
CPS: Combined positive score
ESCC: Esophageal squamous cell carcinoma
FDA: Food and Drug Administration
MHC: Major histocompatibility complex
IFN-γ: Interferon gamma
PFS: Progression-free survival
POC: Proof-of-concept
ORR: Objective response rate
AEs: Adverse events
CT: Computed tomography
RECIST: Response Evaluation Criteria in Solid Tumors
PR: Partial response
CTCAE: Common Terminology Criteria for Adverse Events
